# Systematic Review of Neuropsychiatric Side Effects Induced by Mefloquine: Evidence From Case Reports and Clinical Studies

**DOI:** 10.7759/cureus.115158

**Published:** 2026-08-25

**Authors:** Saravana Kumar Ramasubbu, Ann George, S K Hasib Reja

**Affiliations:** 1 Department of Pharmacology, All India Institute of Medical Sciences (AIIMS), Madurai, Madurai, IND; 2 Department of Pharmacology, Andaman and Nicobar Islands Institute of Medical Sciences (ANIIMS), Pondicherry University, Sri Vijaya Puram, IND

**Keywords:** depress, drug-induced psychosis, mefloquine, mental health illness, neuropsychiatric

## Abstract

Mefloquine is an effective antimalarial drug widely used for malaria treatment and chemoprophylaxis. However, its use has been associated with a range of neuropsychiatric adverse effects, ranging from mild sleep disturbances to severe psychiatric reactions. The aim of this study is to systematically review the available evidence on neuropsychiatric adverse effects associated with mefloquine use and to characterize their clinical spectrum, time to onset, and reported outcomes. A systematic literature search was conducted to identify studies reporting neuropsychiatric adverse effects associated with mefloquine. Studies meeting the predefined eligibility criteria were included, and relevant clinical and adverse effect data were extracted. The methodological quality of included studies was assessed using the National Institutes of Health (NIH) Study Quality Assessment Tools. Of 207 records identified, 25 studies were included after removing duplicates and applying the eligibility criteria. These comprised 17 case reports and 8 clinical studies. The case reports described 19 patients aged 7-63 years with a wide range of neuropsychiatric manifestations. Frequently reported adverse effects included hallucinations, depression, anxiety, and insomnia, while psychosis, confusion, and mania were also reported. Other manifestations included paranoia, delusions, restlessness, nightmares, depersonalization/derealization, suicidal ideation, and delirium. The time to onset ranged from a few hours to several months after exposure. Clinical studies demonstrated variable frequencies of neuropsychiatric adverse effects across different populations and study designs. Mefloquine is associated with a broad spectrum of neuropsychiatric adverse effects, ranging from mild symptoms such as anxiety and insomnia to severe psychiatric manifestations. Early recognition and careful patient selection are important when prescribing mefloquine.

## Introduction and background

Malaria, one of the most severe infectious diseases spread by vectors worldwide, is a major concern to public health, particularly in tropical and subtropical regions. According to estimates from the World Health Organization (WHO), there were 597,000 malaria-related deaths and 263 million cases globally in 2023, with Africa accounting for the majority of cases and deaths. Effective antimalarial medications are still necessary for both treatment and chemoprophylaxis despite significant advancements in malaria prevention and control, especially for tourists, military personnel, and communities living in endemic areas [1]. Chemoprophylactic medications, including artemisinin, chloroquine, and mefloquine, are used in combination therapy to suppress the growth and multiplication of *Plasmodium* sp., the causative agent of various forms of malaria in humans [2]. One of the main antimalarial medications used in both preventative and treatment regimens is mefloquine hydrochloride. It is effective and generally well tolerated, according to extensive research [3]. Mefloquine's extended elimination half-life, easy once-weekly dosage schedule, and shown effectiveness made it a popular chemoprophylactic medication for tourists and military personnel stationed in malaria-endemic regions.

Mefloquine's high lipophilicity facilitates its penetration across the blood-brain barrier, potentially contributing to its propensity for central nervous system and neuropsychiatric adverse effects. A wide range of neuropsychiatric side effects, from minor and transient symptoms to severe and deadly mental illnesses, can be brought on by mefloquine. While more severe reactions such as panic attacks, hallucinations, psychosis, mania, seizures, suicidal ideation, suicide attempts, and completed suicide have also been reported, the most frequently reported symptoms include insomnia, abnormal dreams, anxiety, dizziness, irritability, mood swings, and depression [4,5].

It is still unclear precisely which mechanisms underlie the neuropsychiatric side effects of mefloquine. Mefloquine may interfere with normal brain function in a number of ways, according to several experimental studies. It may lead to GABAergic interneuron dysfunction and an imbalance between excitatory and inhibitory neurotransmission. It also prevents gap junction contact, which hinders direct electrical signaling between neurons. Furthermore, it has been demonstrated that mefloquine inhibits acetylcholinesterase activity and intracellular transport mechanisms, which may have an impact on neuronal signaling and cognitive function. Additionally, experimental data indicate that the medication may cause liver damage and reduce cortical neuronal activity, both of which may indirectly lead to neurotoxicity. The wide range of neuropsychiatric symptoms linked to mefloquine usage, from mild symptoms such as anxiety and insomnia to severe reactions such as psychosis and suicidal conduct, may be explained by these mechanisms, which are probably complex [6].

Neuropsychiatric effects of mefloquine are available from reported case reports and population-based studies. Clinical studies provide information on the frequency and risk of psychiatric adverse effects, whereas case reports offer detailed descriptions of rare, severe, and unusual presentations that may not be captured in larger studies. However, it is difficult to fully understand mefloquine's psychiatric safety profile because this evidence is still scattered across numerous trial types. A systematic review that incorporates data from both case reports and clinical research is necessary in order to synthesize the existing data, characterize patient outcomes, and identify the range of neuropsychiatric side effects.

The review's recommendations will help doctors make informed prescription decisions, promote the early identification and management of psychiatric side effects, and indicate areas that require further investigation.

## Review

Methods

Study Protocol

The Preferred Reporting Items for Systematic Reviews and Meta-Analyses (PRISMA) 2020 standards were followed in the execution and reporting of the review. Prior to starting the review, the systematic review was registered with reference ID number CRD42026142549 in the International Prospective Register of Systematic Reviews (PROSPERO).

Eligibility Criteria

The Population, Exposure, Comparator, and Outcome (PECO) paradigm was used to formulate the eligibility requirements. The population included people of any age or gender who were treated for malaria or given chemoprophylaxis with mefloquine. Mefloquine use, regardless of dosage, duration, or indication, was the exposure of interest. Studies lacking a comparator, such as case reports and case series, were also eligible for inclusion; comparator groups, when available, were placebo, other antimalarial medications, or people not exposed to mefloquine. Mefloquine-related neuropsychiatric side effects, such as anxiety, depression, insomnia, nightmares, panic attacks, mood disorders, hallucinations, psychosis, mania, delirium, suicidal ideation, suicide attempts, completed suicide, and other documented psychiatric manifestations, were the main result.

Inclusion and Exclusion Criteria

Studies on people of any age or gender who received mefloquine for chemoprophylaxis or malaria treatment and reported one or more psychological side effects were included. Clinical studies were suitable for inclusion, including surveys, questionnaire-based investigations, prospective cohort studies, and randomized controlled trials. Published case reports and case series detailing the psychiatric side effects of mefloquine were also included. Studies involving animals and in vitro studies, review articles, duplicate publications, studies that did not report mefloquine-related psychiatric side effects, and studies for which the full text could not be downloaded and only the abstract was available were excluded.

Information Sources and Search Strategy

Three electronic databases, PubMed, Scopus, and Google Scholar, were thoroughly searched from database creation until June 30, 2026, in order to find studies. The following Medical Subject Heading term filters were applied: ("Mefloquine") AND ("Psychiatry" OR "Psychiatric illness" OR "Psychiatric adverse effects" OR "Psychosis" OR "Depression" OR "Anxiety" OR "Insomnia" OR "Nightmares" OR "Hallucinations" OR "Mania" OR "Delirium" OR "Mood Disorders" OR "Suicidal Ideation" OR "Suicide" OR "Mental Disorders"). The detailed literature search strategy is provided in the Appendices.

Data Items, Summary Measures, and Quality Assessment

For each included study, data were extracted using a standardized data extraction form. For clinical studies, the extracted data included the author and year of publication, study population, study design, total sample size, number of participants exposed to mefloquine, comparison group (where applicable), number of participants who developed psychiatric adverse effects in the mefloquine group, and the reported psychiatric manifestations. For case reports and case series, the extracted data included the author and year of publication, patient age, sex, indication for mefloquine use, maximum dose of mefloquine, psychiatric adverse effect(s), time interval between mefloquine administration and the onset of psychiatric adverse effects, psychiatric history, management of psychiatric adverse effects, and symptom remission or clinical outcome. The quality of included studies was assessed using validated Study Quality Assessment Tools from the National Institutes of Health (NIH) [7].

Results

**Figure 1:**
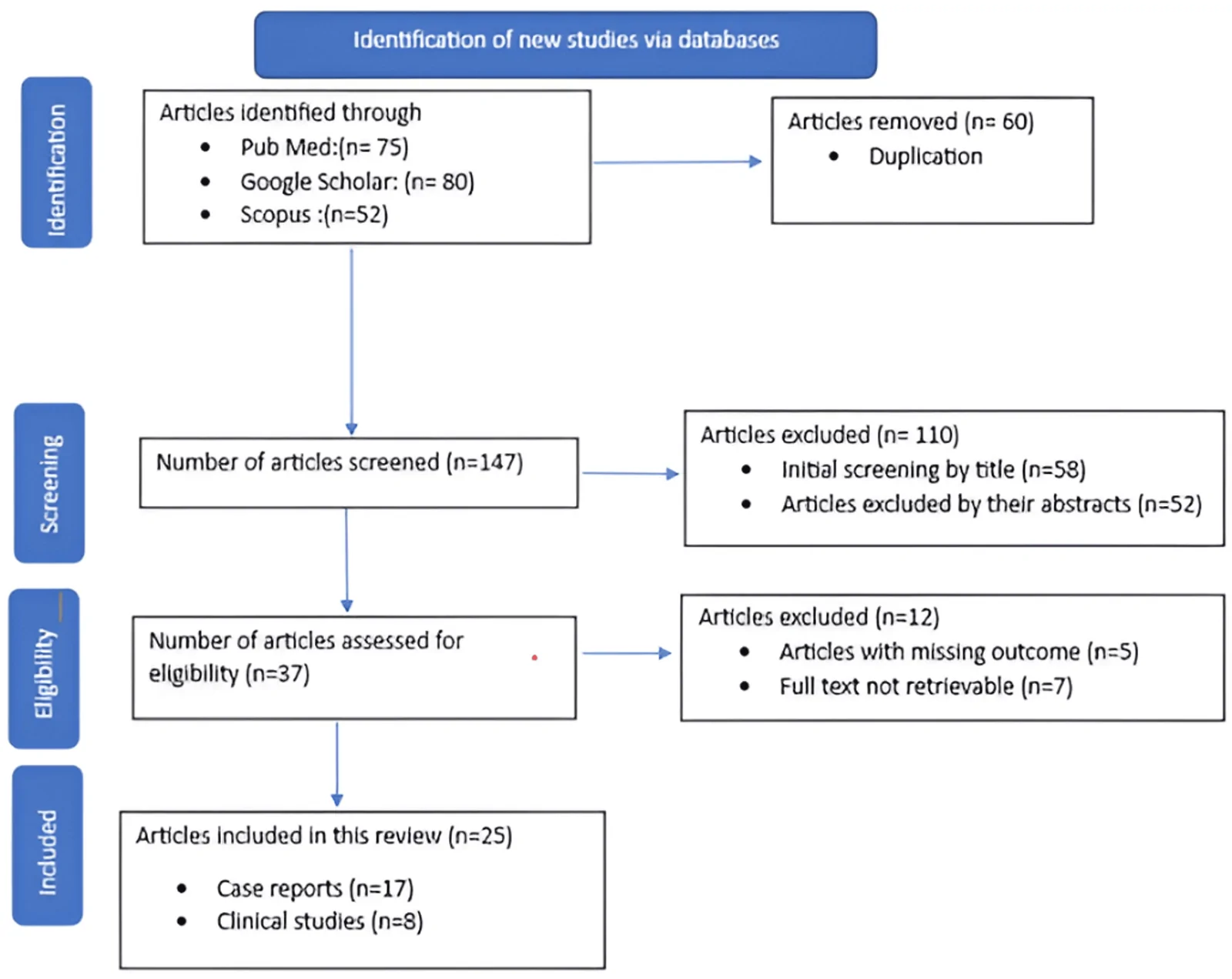
PRISMA flow diagram of selection of studies PRISMA: Preferred Reporting Items for Systematic Reviews and Meta-Analyses

We identified 207 articles (Figure 1), and after removing duplicates and applying inclusion criteria, 25 studies were identified. Among them, 17 were case reports [3,8-23] and 8 were clinical studies [24-31]. The methodological quality of the included studies/risk of bias was assessed using the NIH Study Quality Assessment Tools, and the detailed study-wise quality assessment is presented in the Appendices.

Characteristics of the Included Studies

Twenty-five studies evaluating mefloquine-associated psychiatric adverse effects were included in this systematic review. Seventeen studies were case reports involving individual patients, while eight were clinical studies comprising three randomized controlled trials, two prospective cohort studies, two questionnaire-based studies, and one retrospective telephone survey. The characteristics of the included case reports and clinical studies are summarized in Table 1 and Table 2.

**Table 1:** Characteristics of the included case reports ADR: adverse drug reaction, NR: not reported

Study (year)	Age	Sex	Indication	Maximum dose of mefloquine		Neuropsychiatric ADR	Time frame between medication and neuropsychiatric ADR	Treatment of psychosis	Symptom remission	Psychiatric history
Havaldar and Mogale (2000) [8]	7	Male	Malaria treatment	NR	Hallucination, insomnia		3 days	Withdrawal of mefloquine	Within 24 hours of stopping the drug	No
Thapa and Biswas (2009) [9]	11	Female	Malaria treatment	1,000 mg	Mania and hallucination		NR	Low-dose risperidone	40 days after treatment	No
Stuiver et al. (1989) [10]	Patient 1: 34, patient 2: 56	Patient 1: female, patient 2: male	Malaria treatment	750 mg	Patient 1: delusion, hallucination, confusion, aggressive behavior; patient 2: hallucination, confusion, aggressive behavior		Patient 1: 3 days, patient 2: 2 days	No treatment given	Patient 1: recovered after 2 days of onset of psychosis, patient 2: recovered after 3 days of onset of psychosis	No
Tran et al. (2006) [11]	37	Male	Chemoprophylaxis	250 mg for prophylaxis, 1,250 mg for suspected malaria	Paranoia, agitation		1 day	Haloperidol	15 days after treatment	One episode of panic attack
Dietz and Frölich (2002) [12]	25	Male	Chemoprophylaxis	250 mg for prophylaxis	Paranoid psychosis, depression		8 days after last dose	Haloperidol and lorazepam	24 days after last dose	No
Björkman (1989) [13]	45	Female	Chemoprophylaxis	250 mg for prophylaxis	Acute confusion and depression		4 days after last dose	NR	2 weeks after last dose	No
Oueriagli Nabih et al. (2011) [14]	Patient 1: 31, patient 2: 27	Male	Chemoprophylaxis	250 mg for prophylaxis	Patient 1: mania and psychosis, patient 2: major depressive disorder with psychosis		NR	Clomipramine and olanzapine	Patient 1: after 3 weeks, patient 2: after 4 weeks	No
Peterson et al. (2011) [15]	27	Male	Chemoprophylaxis	250 mg for prophylaxis	Visual hallucinations, restlessness, insomnia		NR	Sertraline and diazepam	NR	No
Brumbaugh et al. (2008) [16]	63	Male	Chemoprophylaxis	250 mg for prophylaxis	Acute confusion, mania, grandiose		10 days after last dose	Lithium, zopiclone	12 days after admission	Yes (mania)
Hennequin et al. (1994) [3]	Patient1: 23, patient 2: 45	Patient 1: male, patient 2: male	Chemoprophylaxis	250 mg for prophylaxis	Patient 1: acute confusion, anterograde memory loss, major agitation; patient 2: melancholic depression with suicidal impulses		Patient 1: 9 days, patient 2: 10 days	Patient 1: sultopride, patient 2: no treatment	Patient 1: 6 days within symptom onset, patient 2: 8 days	No
Javorsky et al. (2001) [17]	52	Female	Chemoprophylaxis	250 mg for prophylaxis	Anxiety, paranoia, visual hallucinations, confusion, depressive symptoms		3 months after last dose	Risperidone, paroxetine	NR	No
McEvoy et al. (2015) [18]	31	Male	Chemoprophylaxis	250 mg for prophylaxis	Depersonalization, derealization		1 week after last dose	Escitalopram, lamotrigine	2 months	No
Clattenburg and Donnelly (1997) [19]	10	Male	Chemoprophylaxis	250 mg for prophylaxis	Anxiety, restlessness		NR	Cognitive and behavioral therapy	NR	No
Tor et al. (2006) [20]	22	Male	Chemoprophylaxis	250 mg for prophylaxis	Visual hallucinations, restlessness, insomnia		2 weeks after last dose	Olanzapine followed by risperidone	2 weeks after last dose	Yes (psychosis)
Yelmo et al. (2010) [21]	26	Female	Chemoprophylaxis	250 mg for prophylaxis	Megalomania, excessive happiness, insomnia		After 6 months of prophylaxis	Risperidone, clonazepam	NR	No
Speich and Haller (1994) [22]	47	Male	Chemoprophylaxis	750 mg	Agitation, delirium, rigors, central anticholinergic syndrome		Within a few hours after last dose	Physostigmine	Within a few hours of administration of physostigmine	No
Even et al. (2001) [23]	50	Male	Chemoprophylaxis	250 mg	Depression, psychosis		After second dose: depression, after 2 weeks: psychosis	Amitriptyline, electroconvulsive therapy, chlorpromazine, carbamazepine	NR	No

**Table 2:** Characteristics of the included clinical studies RCT: randomized controlled trial, ADRs: adverse drug reactions

Study (year)	Study population	Study design	Total sample size (N)	Mefloquine exposed (n)	Number of patients who developed neuropsychiatric ADRs in the mefloquine group (n)	Comparison group	Neuropsychiatric manifestations
van Riemsdijk et al. (2002) [24]	Sample of patients who participated in the MAL30010 trial	RCT	119	58	58	Atovaquone + chloroguanide	Anger, depression
van Riemsdijk et al. (2004) [25]	Patients who consulted the Travel Clinic of the Havenziekenhuis and Institute for Tropical Diseases Rotterdam for mefloquine prophylaxis	Prospective cohort study	151	151	58	None	Insomnia, headache, dizziness, nightmares, anxiety
Corbett et al. (1996) [26]	Returned travellers from malaria-endemic area to Britain	Questionnaire-based survey	255	80	23	None	Depression, anxiety
Barrett et al. (1996) [27]	Travellers who traveled to malaria-endemic area	Retrospective telephonic survey	3,375	1,214	333	None	Hallucination, confusion, depression, anxiety, dissociation from reality, etc.
Overbosch et al. (2001) [28]	Non-immune subjects who traveled to malaria-endemic area	RCT	1,013	477	139	Atovaquone + proguanil	Vivid dreams, insomnia, dizziness, visual difficulties, anxiety, depression
Ringqvist et al. (2015) [29]	Subjects reported to Danish national registry	Questionnaire-based study	73	73	73	None	Anxiety, mania, delusion, somatization, etc.
Sowunmi et al. (1993) [30]	Group of Africans with falciparum malaria treated with mefloquine	Prospective cohort study	317	317	9	None	Irritation talk, titubation of the head, vertigo, hallucination, dizziness, vomiting, etc.
Schlagenhauf et al. (2003) [31]	Non-immune travellers to sub-Saharan Africa	RCT	674	153	118	Chloroquine + proguanil, doxycycline, atovaquone + proguanil	Headache, sleep disturbances

The clinical studies enrolled participants receiving mefloquine for malaria chemoprophylaxis or treatment and included diverse populations such as non-immune travellers, returned travellers, military personnel, patients attending travel clinics, and individuals treated for falciparum malaria. The number of subjects exposed to mefloquine varied from 58 to 1,214 among studies, and sample sizes varied from 73 to 3,375. Comparator groups, where applicable, included atovaquone-proguanil and atovaquone plus chloroguanide, whereas observational studies did not include comparator groups.

Characteristics of the Case Reports

The 17 case reports detailed 19 patients between the ages of 7 and 63 who experienced psychological side effects after being exposed to mefloquine. While some occurrences followed therapeutic applications, the majority happened during malaria chemoprophylaxis. The onset of symptoms varied from a few hours to six months following medication administration. The main management approach involved stopping mefloquine, and a few patients needed psychological care. The majority of patients either fully healed or showed notable clinical improvement.

Spectrum of Psychiatric Adverse Effects

**Figure 2:**
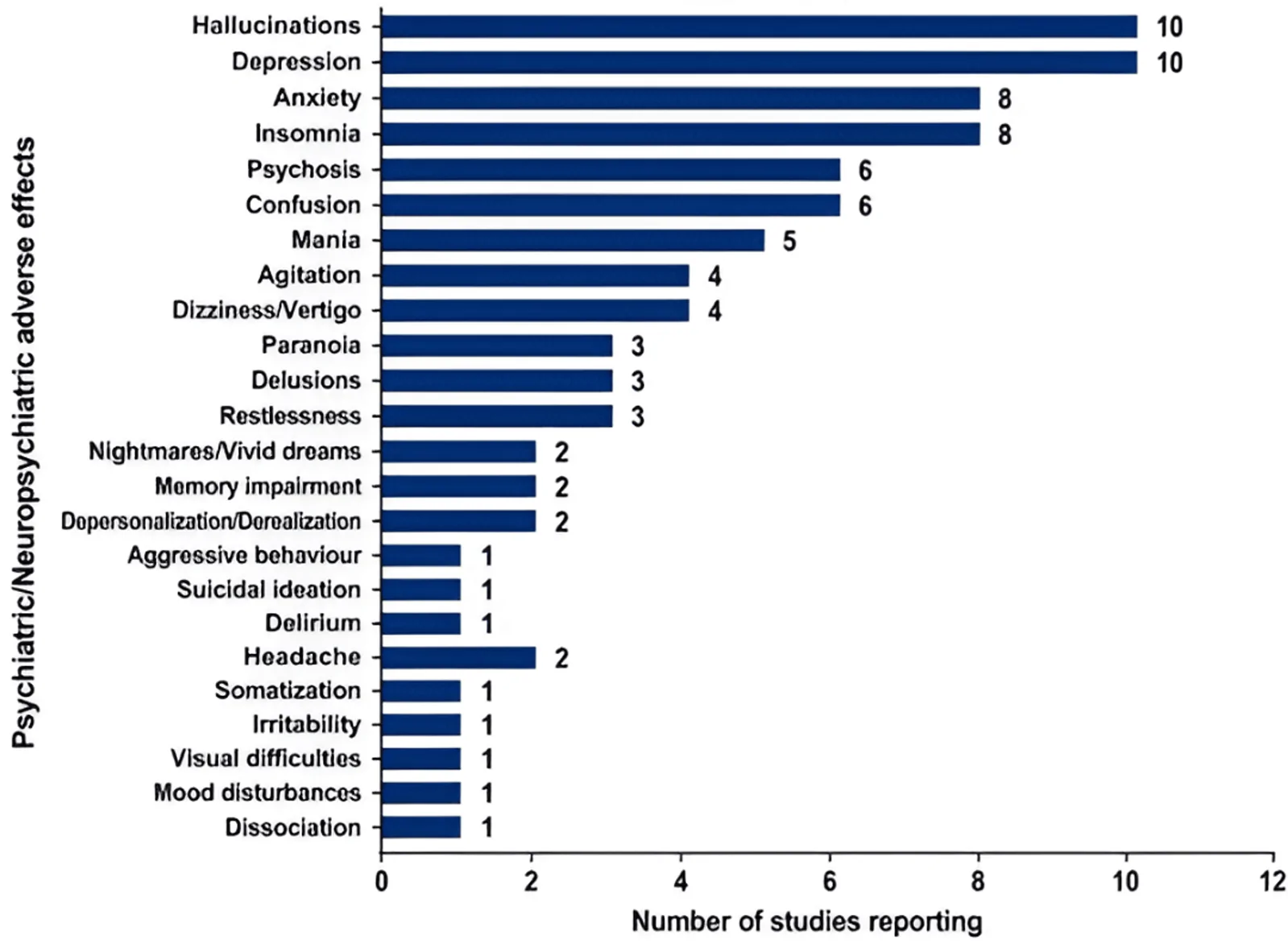
Spectrum of psychiatric and neuropsychiatric adverse effects reported in the included case reports and clinical studies

**Figure 3:**
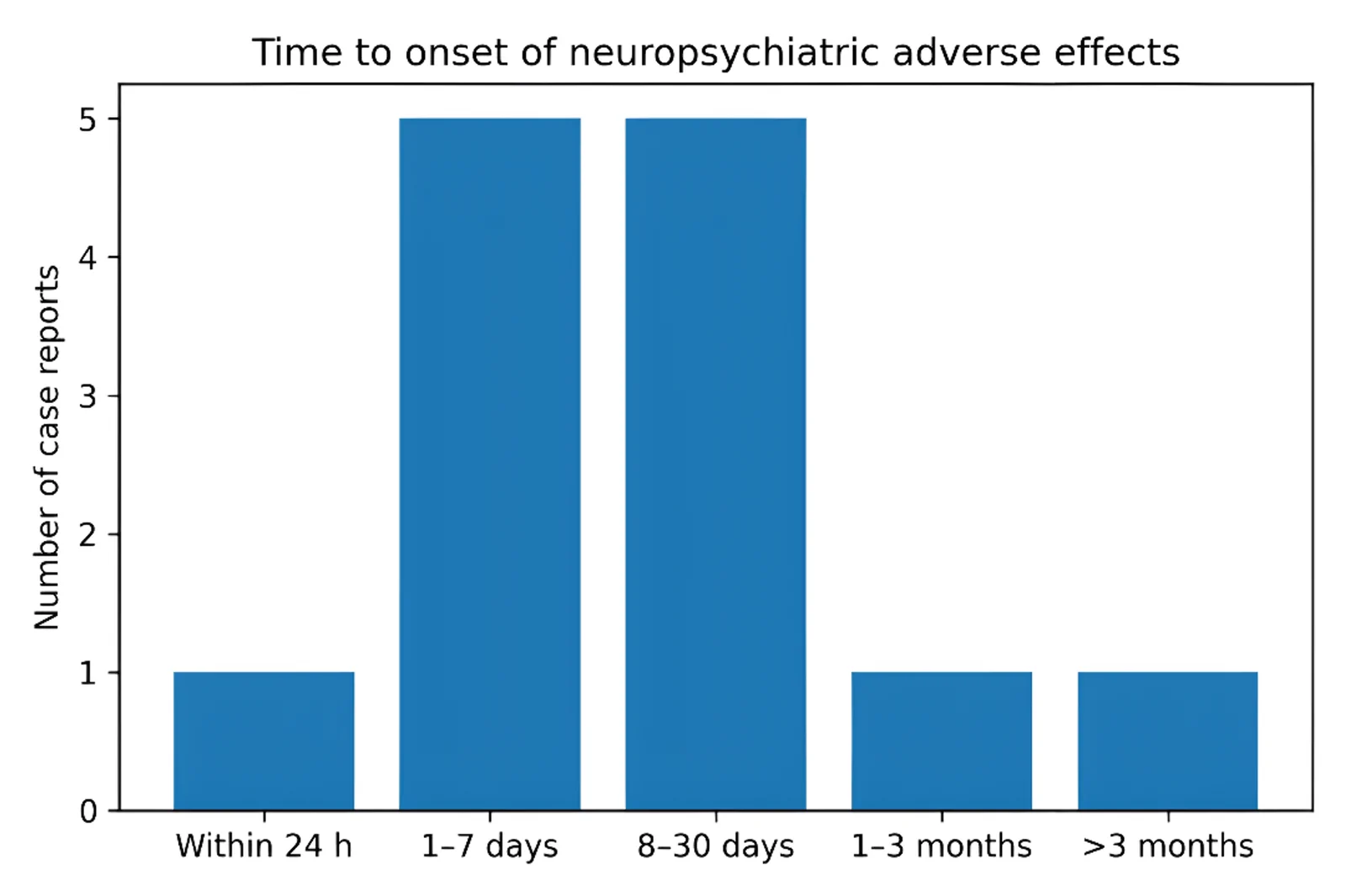
Time to onset of neuropsychiatric adverse effects of case reports

Hallucinations and depression were most frequently reported (10 studies each), followed by anxiety and insomnia (8 studies each). Psychosis and confusion were reported in 6 studies each, with other effects reported less frequently. The distribution of reported neuropsychiatric adverse effects is shown in Figure 2. Neuropsychiatric adverse effects most commonly emerged within 1-30 days, with fewer cases occurring within 24 hours or after 1 month. The distribution of time to onset is shown in Figure 3.

Findings From Clinical Studies

The eight clinical studies assessed the prevalence and trends of mefloquine-related psychiatric side effects in various populations. Anxiety, sadness, insomnia, nightmares, headaches, dizziness, hallucinations, and disorientation were among the side effects that were frequently reported. While observational studies showed varying frequencies based on the study population and evaluation techniques, randomized controlled trials typically found a higher frequency of psychiatric side effects with mefloquine compared with comparator antimalarial medicines.

Discussion

The information on mefloquine-related psychiatric side effects from 25 studies (17 case reports and 8 clinical studies) was included in this systematic review. The findings demonstrate that mefloquine is associated with a broad spectrum of psychiatric adverse effects, ranging from relatively mild manifestations such as anxiety, insomnia, abnormal dreams, and depression to severe psychiatric disorders including psychosis, hallucinations, mania, delirium, and suicidal ideation. Although severe reactions were predominantly described in case reports, clinical studies consistently reported a higher frequency of neuropsychiatric adverse effects among individuals receiving mefloquine compared with comparator antimalarial regimens. Together, these findings reaffirm that neuropsychiatric toxicity remains one of the most clinically important adverse effects limiting the use of mefloquine.

The present review reveals considerable variation in the clinical manifestation of psychiatric side effects linked to mefloquine. Depression, hallucinations, anxiety, insomnia, psychosis, confusion, and mania were the most frequently reported manifestations, whereas aggression, paranoia, depersonalization, derealization, memory impairment, and suicidal ideation were reported less frequently. In double-blind studies, 29%-77% of mefloquine users at preventive doses of 250 mg weekly experience a variety of neuropsychiatric side responses, including unusual or vivid nightmares, dizziness, vertigo, cognitive impairment, anxiety, and sadness [28,31].

An important observation of this review is the marked variability in the time to onset of psychiatric adverse effects. Although most patients developed symptoms within the first week of mefloquine administration, several reports documented delayed onset occurring weeks or even months after exposure. Similar observations have been reported previously, indicating that neuropsychiatric adverse effects may occur during treatment, after repeated dosing, or occasionally after drug discontinuation [4,32]. The prolonged elimination half-life of mefloquine, which ranges from approximately two to four weeks, may partly explain the persistence or delayed appearance of psychiatric symptoms in susceptible individuals [33]. Therefore, during therapy and the post-exposure phase, clinicians should be on the lookout, especially for patients who exhibit early neuropsychiatric symptoms.

The evidence that mefloquine causes psychiatric side effects is strengthened by the results of the included clinical studies. In general, randomized controlled trials showed that those taking mefloquine experienced more neuropsychiatric adverse effects than those taking other antimalarial medications, such as atovaquone-proguanil. Similarly, depression, anxiety, insomnia, nightmares, dizziness, and mood disorders were often reported side effects in observational studies involving travellers and military personnel. The consistency of results across several study designs shows a causal relationship between mefloquine exposure and neuropsychiatric toxicity, although the reported frequencies varied significantly due to variations in study demographics, outcome assessment techniques, and follow-up duration.

The best comparison evidence comes from randomized studies. In particular, van Riemsdijk et al. [24] assessed neuropsychiatric adverse events in mefloquine users in comparison to atovaquone with chloroguanide and found clinically significant variations in neuropsychiatric outcomes. Similarly, Overbosch et al. [28] compared atovaquone-proguanil with mefloquine in a randomized, double-blind study of non-immune travellers and reported neuropsychiatric manifestations including vivid dreams, insomnia, dizziness, visual difficulties, anxiety, and depression among the outcomes assessed. The Schlagenhauf randomized trial provides an important nuance [31]. Although many adverse events were reported, none were serious, and the study did not demonstrate a significant difference in overall adverse event frequency between treatment groups. However, mefloquine users had an increased occurrence of moderate neuropsychological adverse effects in subgroup analyses. Thus, the available randomized evidence does not imply that every psychiatric outcome is consistently more frequent with mefloquine, but it does support a clinically relevant neuropsychological adverse effect profile. Observational studies similarly identified a range of psychiatric symptoms. Barrett et al. [27] reported hallucinations, confusion, depression, anxiety, and dissociation from reality, while Corbett et al. [26] identified depression and anxiety among returned travellers. Ringqvist et al. [29] reported anxiety, mania, delusions, and somatization in a Danish adverse event population. These findings demonstrate that the spectrum identified in the case reports is also represented in larger observational populations.

Several factors may influence susceptibility to mefloquine-associated psychiatric adverse effects, although the evidence remains inconsistent. Previous psychiatric illness is the most clinically relevant consideration. Severe reactions have been reported both in individuals with no previous psychiatric history and in patients with pre-existing psychiatric disorders, indicating that psychiatric history alone does not explain all cases. Body weight or body mass index (BMI) may also influence susceptibility. One of the included prospective studies reported that a low BMI was associated with an increased risk of neuropsychiatric adverse events and concentration impairment in women receiving mefloquine [25]. Similarly, another included study noted that women taking mefloquine reported more psychiatric adverse effects than other drugs [31]. This finding suggests that individual pharmacokinetic differences may contribute to susceptibility, although it requires confirmation in larger populations.

From a clinical perspective, the review's findings highlight how important it is to properly select patients before administering mefloquine. A complete psychiatric history should be obtained in order to ascertain whether a patient has a history of depression, anxiety disorders, psychosis, or other mental problems that could render them more susceptible to adverse outcomes. The possibility of early neuropsychiatric symptoms, such as insomnia, anxiety, strange nightmares, mood swings, or behavioral changes, should be sufficiently discussed with patients. Additionally, they should be told to cease taking the medicine as quickly as possible and to get help if these symptoms appear. Early detection, stopping mefloquine, and receiving proper mental healthcare resulted in complete recovery or notable clinical improvement in most of the recorded cases included in this study.

The main limitation of the study was the predominance of case reports, which introduces publication or reporting bias. Direct comparisons between studies were hampered by significant variation in study populations, designs, outcome definitions, and adverse effect assessment techniques. Recall and reporting bias may have been introduced in certain studies that used self-reported or retrospective data. Notwithstanding these drawbacks, the overall evidence for a clinically significant link between mefloquine exposure and psychiatric side effects is strengthened by the consistency of neuropsychiatric manifestations across various study designs and groups.

## Conclusions

Mefloquine is associated with a broad spectrum of neuropsychiatric adverse effects, ranging from anxiety and insomnia to severe reactions such as hallucinations, psychosis, and mania. To reduce any harm, early detection and suitable patient selection are crucial. To further understand the prevalence, risk factors, and long-term consequences of these negative impacts, more prospective research is required.

## Appendices

Table 3 presents the detailed search strategy used in the present study. 

**Table 3:** Search strategy

Database	Search string
PubMed	("Mefloquine"[MeSH Terms] OR ("Mefloquine"[Supplementary Concept] OR "Mefloquine"[All Fields] OR "mefloquin"[All Fields] OR "Mefloquine"[MeSH Terms])) AND ("Mental Disorders"[MeSH Terms] OR "Drug-Related Side Effects and Adverse Reactions"[MeSH Terms] OR "psychiat*"[All Fields] OR ("psychiatrical"[All Fields] OR "psychiatrically"[All Fields] OR "psychiatrics"[All Fields] OR "psychiatry"[MeSH Terms] OR "psychiatry"[All Fields] OR "psychiatric"[All Fields]) OR ("psychotic disorders"[MeSH Terms] OR ("psychotic"[All Fields] AND "disorders"[All Fields]) OR "psychotic disorders"[All Fields] OR "psychosis"[All Fields]) OR ("depressed"[All Fields] OR "depression"[MeSH Terms] OR "depression"[All Fields] OR "depressions"[All Fields] OR "depression s"[All Fields] OR "depressive disorder"[MeSH Terms] OR ("depressive"[All Fields] AND "disorder"[All Fields]) OR "depressive disorder"[All Fields] OR "depressivity"[All Fields] OR "depressive"[All Fields] OR "depressively"[All Fields] OR "depressiveness"[All Fields] OR "depressives"[All Fields]) OR ("anxiety"[MeSH Terms] OR "anxiety"[All Fields] OR "anxieties"[All Fields] OR "anxiety s"[All Fields]) OR ("insomnia s"[All Fields] OR "sleep initiation and maintenance disorders"[MeSH Terms] OR ("sleep"[All Fields] AND "initiation"[All Fields] AND "maintenance"[All Fields] AND "disorders"[All Fields]) OR "sleep initiation and maintenance disorders"[All Fields] OR "insomnia"[All Fields] OR "insomnias"[All Fields]) OR "nightmare*"[All Fields] OR "hallucination*"[All Fields] OR ("mania"[MeSH Terms] OR "mania"[All Fields] OR "manias"[All Fields]) OR ("suicidal ideation"[MeSH Terms] OR ("suicidal"[All Fields] AND "ideation"[All Fields]) OR "suicidal ideation"[All Fields]) OR ("suicid"[All Fields] OR "suicidal ideation"[MeSH Terms] OR ("suicidal"[All Fields] AND "ideation"[All Fields]) OR "suicidal ideation"[All Fields] OR "suicidality"[All Fields] OR "suicidal"[All Fields] OR "suicidally"[All Fields] OR "suicidals"[All Fields] OR "suicide"[MeSH Terms] OR "suicide"[All Fields] OR "suicides"[All Fields] OR "suicide s"[All Fields] OR "suicided"[All Fields] OR "suiciders"[All Fields]) OR ("delirium"[MeSH Terms] OR "delirium"[All Fields] OR "delirium s"[All Fields] OR "deliriums"[All Fields]) OR (("affect"[MeSH Terms] OR "affect"[All Fields] OR "mood"[All Fields]) AND "disorder*"[All Fields]))
Scopus	TITLE-ABS-KEY (mefloquine) AND TITLE-ABS-KEY ("neuropsychiatric" OR "psychiatric" OR "psychological" OR "neurological" OR "mental" OR psychosis OR hallucination* OR anxiety OR depression OR "suicidal ideation" OR "suicidal behavior" OR insomnia OR "abnormal dreams" OR "sleep disturbance*" OR "adverse effect*" OR "adverse event*" OR toxicity)
Google Scholar	"mefloquine" AND ("neuropsychiatric" OR psychiatric OR psychological OR neurological OR psychosis OR hallucination* OR anxiety OR depression OR "suicidal ideation" OR "suicidal behavior" OR insomnia OR "abnormal dreams" OR "sleep disturbance*" OR "adverse effects" OR "adverse events" OR toxicity)

 Table 4 presents the quality assessment of the included studies.

**Table 4:** Overall methodological quality assessment of the included studies using the NIH Study Quality Assessment Tools NIH: National Institutes of Health

Study	Design	Overall quality	Justification
Havaldar and Mogale (2000) [8]	Case report	Good	Clear exposure, adverse effects, temporal relationship, withdrawal, and outcome reported
Thapa and Biswas (2009) [9]	Case report	Good	Clinical presentation, exposure, and treatment/outcome adequately reported
Stuiver et al. (1989) [10]	Case report (2 patients)	Fair	Clinical manifestations and recovery reported, but several details are limited
Tran et al. (2006) [11]	Case report	Good	Exposure, onset, manifestations, treatment, and outcome described
Dietz and Frölich (2002) [12]	Case report	Good	Exposure history, manifestations, treatment, and outcome reported
Björkman (1989) [13]	Case report	Fair	Clinical presentation and outcome reported; diagnostic/management details limited
Oueriagli Nabih et al. (2010) [14]	Case report	Fair	Case description available, but follow-up and causality details limited
Peterson et al. (2011) [15]	Case report	Good	Psychiatric presentation, exposure history, and clinical course reported
Brumbaugh et al. (2008) [16]	Case report	Good	Detailed clinical description and outcome; psychiatric history reported
Hennequin et al. (1994) [3]	Case report (2 patients)	Fair	Severe manifestations described; long-term follow-up limited
Javorsky et al. (2001) [17]	Case report	Fair	Clinical presentation reported; confounding/follow-up information limited
McEvoy et al. (2015) [18]	Case report	Good	Clinical course, management, and outcome adequately described
Clattenburg et al. (1997) [19]	Case report	Fair	Case presentation described; follow-up/supporting details limited
Tor et al. (2006) [20]	Case report	Good	Psychiatric manifestations and clinical course clearly described
Yelmo et al. (2010) [21]	Case report	Fair	Case presentation reported; follow-up information limited
Speich and Haller (1994) [22]	Case report	Fair	Case presentation reported; follow-up information limited
Even et al. (2001) [23]	Case report	Fair	Manifestations and treatment reported; outcome/causality information limited
van Riemsdijk et al. (2002) [24]	RCT	Good	Randomized comparative design with defined comparator and outcomes
van Riemsdijk et al. (2004) [25]	Prospective cohort	Good	Prospective design with defined exposed population and outcome assessment
Corbett et al. (1996) [26]	Questionnaire survey	Fair	Self-reported questionnaire outcomes with potential recall/reporting bias
Barrett et al. (1996) [27]	Retrospective telephone survey	Fair	Retrospective self-reported data with potential recall/response bias
Overbosch et al. (2001) [28]	RCT	Good	Randomized comparative design with active comparator and reported outcomes
Ringqvist et al. (2015) [29]	Questionnaire study	Fair	Questionnaire-/registry-based outcomes with potential reporting bias
Sowunmi et al. (1993) [30]	Prospective cohort	Good	Prospective cohort with defined treatment exposure and outcome assessment
Schlagenhauf et al. (2003) [31]	RCT	Good	Randomized comparative study with multiple comparator regimens and reported outcomes

Study category	Total	Good	Fair
Case reports	17	8	9
Clinical studies	8	5	3
Overall	25	13	12
